# CAA-YOLO: Combined-Attention-Augmented YOLO for Infrared Ocean Ships Detection

**DOI:** 10.3390/s22103782

**Published:** 2022-05-16

**Authors:** Jing Ye, Zhaoyu Yuan, Cheng Qian, Xiaoqiong Li

**Affiliations:** 1 School of Life Science, Beijing Institute of Technology, Beijing 100081, China; wait_fof@163.com (J.Y.); chengqc9512@foxmail.com (C.Q.); 2 Key Laboratory of Convergence Medical Engineering System and Healthcare Technology, Ministry of Industry and Information Technology, Beijing 100081, China; sam_yuan1960@163.com; 3 School of Medical Technoloy, Beijing Institute of Technology, Beijing 100081, China

**Keywords:** small targets detection, combined attention mechanism, multiscale feature fusion, infrared image, multiscale objects

## Abstract

Infrared ocean ships detection still faces great challenges due to the low signal-to-noise ratio and low spatial resolution resulting in a severe lack of texture details for small infrared targets, as well as the distribution of the extremely multiscale ships. In this paper, we propose a CAA-YOLO to alleviate the problems. In this study, to highlight and preserve features of small targets, we apply a high-resolution feature layer (P2) to better use shallow details and the location information. In order to suppress the shallow noise of the P2 layer and further enhance the feature extraction capability, we introduce a TA module into the backbone. Moreover, we design a new feature fusion method to capture the long-range contextual information of small targets and propose a combined attention mechanism to enhance the ability of the feature fusion while suppressing the noise interference caused by the shallow feature layers. We conduct a detailed study of the algorithm based on a marine infrared dataset to verify the effectiveness of our algorithm, in which the AP and AR of small targets increase by 5.63% and 9.01%, respectively, and the mAP increases by 3.4% compared to that of YOLOv5.

## 1. Introduction

Scene understanding based on machine vision is a key technology in the field of marine transportation, and object detection has received extensive attention as an important part of it. Infrared imaging has been widely used in marine ship detection because of its outstanding characteristics of strong anti-interference ability and all-weather operation [1]. Different from other detection tasks, the ocean scene has its unique characteristics: (1) the scenes including multiscale targets have a broader field of view; (2) the size of targets is extremely distributed. At the same time, due to the low signal-to-noise ratio and poor spatial resolution of infrared images, the visual effect is fuzzy and the target is seriously lacking in texture details, especially for small objects, which bring great challenges to the detection of marine targets based on infrared scenes.

Object detection methods based on deep learning have been shown to greatly improve the performance of object detection. There exist a sea of detection frameworks such as two-stage algorithms (R-CNN [2], fast R-CNN [3], faster R-CNN [4]) and one-stage algorithms (YOLO [5,6,7], SSD [8]). However, it has always been a difficult point of research to improve the detection of small targets while ensuring the detection of large and medium targets. To solve this problem, a series of methods such as multiscale feature fusion strategies and context learning have been proposed. Nayan et al. [9] proposed to use upsampling and skip connections to extract the multiscale features of different network depths during the training process. Deng et al. [10] proposed an extended feature pyramid network, which uses an additional high-resolution pyramid level specifically for small object detection. Tan et al. [11] proposed a weighted bidirectional feature network named BiFPN for adaptively selecting the importance of different features in the fusion process. Lim et al. [12] proposed a method using context to connect multiscale features, supplemented by an attention mechanism to focus on the target in the image. Zhang [13] et al. proposed to use a feature fusion network to comprehend different levels of convolutional features and better utilize the fine-grained features and semantic features of targets.

In the infrared-based ocean scene, the difficulty of object detection is further aggravated by the huge change of ship scale and the weak texture features of small targets. Therefore, directly introducing an extra high-resolution pyramid level brings more noise in the bottom of the feature fusion stage. The use of a traditional backbone network could easily lead to feature degradation or even disappearance of extremely small targets in the process of downsampling and the context feature fusion will be extracted deficiently without guidance.

Based on the characteristics of infrared ocean ship scenes, we propose a CAA-YOLO infrared ship detection algorithm based on one-stage detector YOLOv5. As a state-of-the-art detector, YOLOv5 has the advantages of fast convergence, high accuracy and lightweight model. It also has strong real-time image detection capabilities and low hardware computing requirements, which means it is easy to be transplanted to mobile devices for application in marine traffic scenarios. In the design of the CAA-YOLO network, to better detect small and weak objects, we add the high-resolution feature layer P2 to obtain more shallow details and location information. In order to suppress shallow noise caused by the P2 layer and enhance the feature extraction ability, we introduce the attention module TA into the backbone. In the feature fusion stage, we construct a new feature connection mode to offer more contextual information for the shallow layer. Moreover, we design the combined attention mechanism to achieve adaptive feature fusion, which not only enhances the semantic information of targets but also suppresses the noise interference caused by the P2 layer. We use infrared ship dataset to evaluate the performance of our method. Compared with the baseline network YOLOv5, CAA-YOLO improves the detection performance of small targets in extreme multiscale scenes. Especially for scenes with small targets and intensive and mutual occlusion, in which pixel areas are less than 32 × 32, its AP and AR increase by 5.63% and 9.01%, respectively. In addition, the mAP of all ships increases by 3.4%. The main contributions of the research can be summarized as follows:

- Aiming at the problems existing in infrared ocean ship scenes, this paper proposes a CAA-YOLO for infrared ocean ship detection based on YOLOv5. By introducing an attention module in the stage of feature extraction and feature fusion to utilize more shallow information, we improve the detection for small and weak targets. Compared with some state-of-the-art algorithms, the proposed method achieves better detection results.
- To reserve more shallow details and location information, we add the high-resolution feature layer P2, which improves the detection accuracy of small objects.
- To suppress the background noise and allow the network to independently distinguish the correlation and effectiveness between different feature mapping channels, we introduce a TA module into the backbone network.
- To capture the long-range contextual information of small objects, we design a novel feature fusion method and use a combined attention mechanism to enhance the ability of feature fusion and suppress the noise interference brought by shallow feature layers.

## 2. Related Work

**Figure 1:**
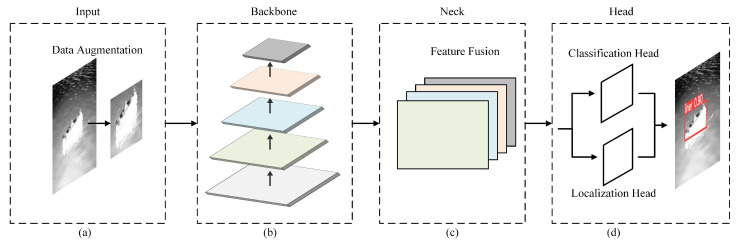
Modern detection structures with the input, backbone, neck and head. (**a**) Input: data preprocessing modules, such as data augmentation; (**b**) Backbone: feature extraction module; (**c**) Neck: multiscale feature fusion module; (**d**) Head: object classification and localization module.

Object detection technology has made remarkable progress due to the development of deep learning technology in recent years. Currently, popular object detection algorithms can be divided into two categories: two-stage algorithms represented by RCNN [2] and faster RCNN [4], and one-stage algorithms represented by YOLO [5] and SSD [8]. They mainly contain input, backbone, neck and head modules as shown in Figure 1. Small targets have fewer pixels than conventional objects, so it is difficult to extract better features in multiscale object detection algorithms. Moreover, with the increasing depth of the convolutional neural network, the details and location features of small targets are gradually lost. In this section, firstly, we briefly review the major works of target detection when adding a high-resolution feature layer, enhancing the feature extraction ability of the backbone network and multiscale feature fusion. Secondly, we briefly introduce the development status of an infrared small target detection.

With higher resolution and more detailed information for localization, low-level features maps are particularly important for small target detection. Adding the shallow characteristics of the high-resolution layer has become one of the methods for small target detection. That is, the shallow feature map in the backbone module of Figure 1b is integrated into the neck module of Figure 1c. Kim et al. [14] proposed the structure of ECAP-YOLO to improve the detection performance of small targets in aerial photography scenes. Shao et al. [15] proposed an adaptive spatial feature fusion network with a high-resolution detection layer to enhance the effect of ship detection in night remote sensing scenes.

As a shared structure of different neural networks, the backbone is the main element of the model, which determines the basic performance. To extract the feature information more effectively, an attention mechanism is widely used in convolution neural networks, which is usually introduced into the modules of the backbone as shown in Figure 1b. Bi et al. [16] embedded a visual attention enhancement network into the backbone network DSOD to extract visual features, which improved the performance of ship detection. Cui et al. [17] proposed a spatial mixing group enhancement (SSE) attention module into the backbone network to suppress some noise while extracting stronger semantic features to reduce false positives caused by inshore and inland interference. Chen et al. [18] designed a novel and lightweight extended attention module (DAM) to extract the discriminant features of ship targets. The integrated attention mechanism suppressed irrelevant regions and highlighted salient features that were useful for ship detection tasks.

One of the main difficulties of object detection is the effectiveness of feature representation with multiscale information processing in the feature fusion stage shown in Figure 1c. Some researchers tried to improve the multiscale feature expression ability. Dewi et al. [19] applied SPP to collect the local region features at different scales in the same CNN layer to learn multiscale object features more comprehensively. Liu et al. [20] proposed a novel RF block (RFB) module, which took the relationship between the size and eccentricity of RFs into account, to enhance the feature discriminability and robustness. Some researchers studied the connection mode of the feature map for a better feature fusion. Liu et al. [21] proposed the structure of FPN, introduced bottom-up and top-down network structure and fused the features of adjacent layers to achieve feature enhancement. Liu et al. [22] proposed PANet, which added an additional bottom-up path aggregation on the basis of FPN. Zhou et al. [23] proposed a scale transfer module to take advantage of cross-scale features. YOLO-V4-lightship [24] has greatly reduced the number of convolutional layers in CSPDarknet53 and used a bottom-up information fusion to improve the precise positioning of ship detection. In addition, an adaptive feature fusion method was also studied to improve the expressive ability of multiscale features. Tan et al. [11] proposed a simple and efficient bidirectional feature pyramid network named BiFPN, which introduced learnable weights to learn the importance of different input features. Hu et al. proposed PAG-YOLO [25] with attention mechanisms in spatial and channel dimensions to adaptively assign the importance of features at different scales.

In addition to the above methods, some other improvement strategies have been put forward in recent years. Researchers have successively proposed loss functions such as GIoU-Loss [26], CIoU-Loss, DIoU-Loss [27] based on IoU to solve the target regression problem. In order to solve the problem of target imbalance, Kisantal [28] et al. proposed a replication enhancement method to increase the number of training samples for small targets; Chen [29] et al. proposed an adaptive resampling strategy by considering target context information to solve the target background mismatch caused by direct copy and paste. In terms of training deep neural networks, the Harris hawks optimization (HHO) [30] algorithm was proposed to tune the hyperparameters of a CNN, which attained 100% accuracy for hand gesture classification. Ref. [31] proposed a simple warm restart technique for stochastic gradient descent to improve its anytime performance.

With the rapid development of deep learning object detection research, some related works based on deep learning have also appeared in the field of infrared small target detection, as is shown in Table 1. In terms of optimizing the backbone network structure, Lin et al. [32] proposed a seven-layer end-to-end convolutional neural network, in which the network structure did not perform image downsampling operations to ensure the accuracy of target localization. M. Li et al. [33] proposed SE-YOLO, a real-time pedestrian object detection algorithm for small objects in infrared images, which improves the feature modeling ability of the network by introducing an SE block [34] into YOLOv3, which improved the feature expression ability of the network combined with the SE block. Li et al. [35] developed a detector, YOLO-ACN, by introducing an attention module and a depth-wise separable convolution. Sun et al. [36] proposed I-YOLO, which modified the backbone with EfficientNet and added a preposition network, DRUNet, to reduce the noise of infrared images. Dai et al. [37] put forward a novel object detection approach, termed TIRNet, where the residual branch was introduced to get robust and discriminating features for accurate box regression and classification. Du et al. [38] proposed FA-YOLO with a CBAM module in the backbone to enhance the performance of infrared occlusion object detection under a confusing background. In terms of improving feature fusion strategy, Dai et al. [39] proposed the asymmetric contextual modulation (ACM), which explored the fusion method between deep and shallow features. Inspired by the improved networks of UNet [40,41,42,43], a dense nested attention network (DNANet) [44] was proposed to resolve the detection of infrared small targets with dense nested connection and a CSAM attention mechanism. Cao et al. [45] presented ThermalDet, which included a dual-pass fusion block (DFB) and a channel-wise enhancement module (CEM) to enhance the performance in thermal imagery. In terms of adding a high-resolution small target detection layer, Li et al. [1] proposed a region-free object detector named YOLO-FIRI for infrared (IR) images by compressing channels and optimizing parameters. To maximize the use of shallow features, the cross-stage-partial-connections (CSP) module in the shallow layer was changed and an improved attention module was introduced into the residual blocks.

These methods achieved a better performance for object detection in different fields, such as pedestrian detection and autonomous driving, but they cannot be directly applied to the detection of infrared ocean ships due to the severe distribution of target sizes in the ocean scene, the weak characteristics of small targets and the interference of noise. Therefore, this paper studied the state-of-the art detector YOLOv5; we applied a high-resolution feature layer so that shallow details and location information can be better used. To suppress shallow noise, we introduced the TA module into the backbone. Besides, we utilized a new feature fusion method to capture long-range contextual information for small targets and designed a combined attention mechanism to enhance the ability of feature extraction and feature fusion while suppressing the noise interference caused by the shallow feature layers.

## 3. Methodology

Aiming at the severe distribution of ocean ship size and weak small target features in infrared scenes, we propose a combined-attention-augmented YOLO (CAA-YOLO) based on YOLOv5. In this section, we describe the overall framework of the proposed method and three specific improvement measures in detail. A shallow feature layer P2 is added to the structure of FPN, then we build the backbone enhancement module TA and feature fusion module CAA based on the P2 layer with multiple attention mechanisms.

### 3.1. Network Architecture

**Figure 2:**
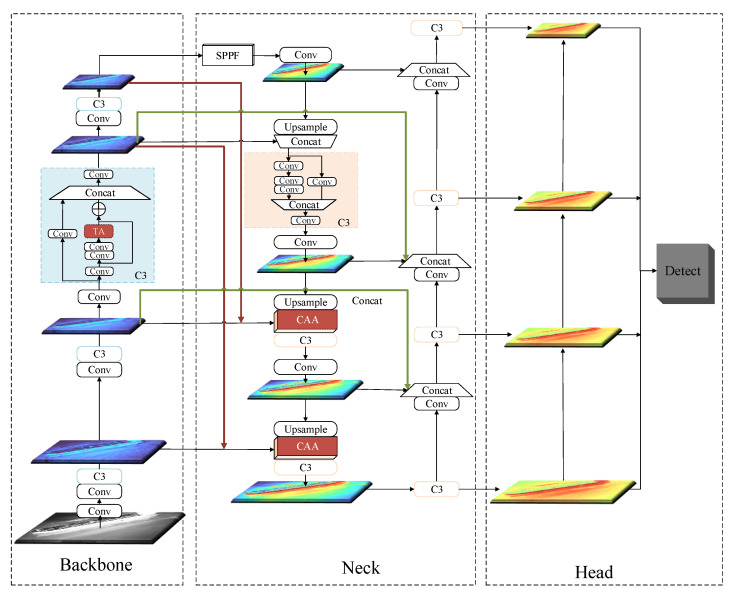
The framework of the CAA-YOLO.

Figure 2 shows the overall framework of the proposed method. YOLOv5 consists of four main modules. The first is the input module, in which data are preprocessed for given input images. YOLOv5 uses mosaic data enhancement, automatic calculation of anchor boxes and image scaling to process input images. The second is the backbone module, where CSPDarknet53 is used as the backbone network to perform feature extraction on images. The third is the neck module, the path aggregation network [22] (PANet), a cyclic pyramid structure composed of convolution operations, upsampling operations and CSP2_X, which performs feature fusion operations on multiscale feature maps from the backbone network. The fourth is the head module, which mainly performs the operations of target regression and localization.

CAA-YOLO is mainly improved for the backbone and neck modules: (1) To make full use of the location and details information of small targets provided by the underlying feature layer, the feature map P2 extracted by the backbone module is fused to the neck module to make the network adaptable to infrared ship targets with extreme scale changes. (2) To extract more effective feature information, we introduce the triplet attention [47] mechanism into the residual block of the backbone to help the model better extract features of interesting targets and suppress noise interference. (3) To prevent the noise brought by the feature map P2 from affecting the entire feature fusion stage and provide more contextual information for small targets, the feature connection method of the neck module is modified. Besides, we use a hybrid attention module to guide the feature fusion process to make the feature fusion more effective.

### 3.2. High-Resolution Feature Layer P2

As the pixel proportion of small targets is small, the feature information is generally reduced after several downsampling layers in the process of feature extraction by the convolutional neural network. For example, when the step size stride is 16, a target region with 32 × 32 pixels is only 2 × 2 pixels in the feature map, and the effective regions for detecting small objects cannot be discerned. In addition, with the deepening of the network layers, the feature information and location information of small targets are gradually lost, which is not conducive to the localization of the target [48,49]. In the convolutional neural network, the high-level feature map has a large receptive field and rich semantic features, but it loses a lot of spatial features. On the contrary, the shallow feature map has a small receptive field, but it has high spatial resolution and accurate target locations, which is suitable for small object detection [50].

**Figure 3:**
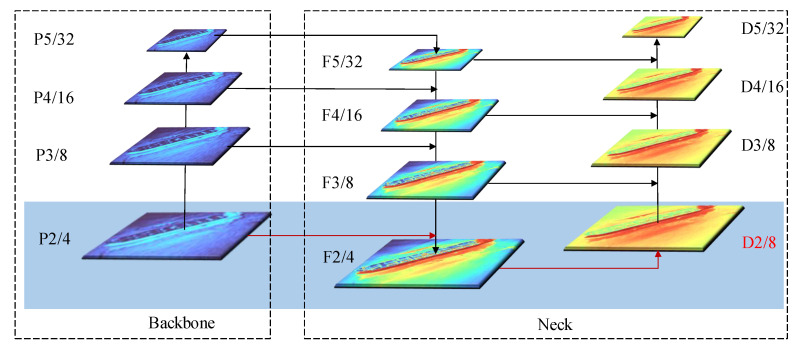
Structure with high-resolution detection layer P2. Red lines represent the newly added P2 layer.

The backbone network is a convolutional neural network that extracts feature maps of different sizes from input images through multiple convolution layers and pooling layers. YOLOv5 uses 3 scales of output feature maps to detect objects of different sizes and 8-time downsampled feature maps to detect small objects. However, there are a large number of small targets in the datasets of the infrared ocean, and the area distribution is shown in Table 2. For tiny targets with less than 10 × 10 pixels of the size, the target features compressed by 8-time downsampling are extremely weak and may even disappear completely, so we added a high-resolution feature map with a 4-time downsampling to detect tiny targets, its structure is shown in Figure 3. First, the feature map P2 extracted by the backbone network is fused with feature maps of other scales through FPN [21] and PAN [22] structures. Then, a D2 detection head dedicated to tidying target detection is constructed using the fused F2 features, so that the network can utilize more location information and feature information of small objects provided by the high-resolution feature layer P2. Mathematically, the D2 layer can be obtained by Formula (1):

(1) $$\begin{array}{c} D2=\mathrm{Concat}\left[P2,\mathrm{Upsampling}\left(\mathrm{Conv}\left(C3\left(F3\right)\right)\right)\right] \end{array}$$

C3(I) represents the feature map I as the input data of the C3 module, Conv(I)) represents the convolution operation on the input I, Upsampling(I) represents the double upsampling operation on input I, Concat(I1, I2) indicates that the input features I1 and I2 are stitched according to the channel.

### 3.3. Enhanced Backbone

Ocean targets based on infrared scenes contain a lot of noise. If the shallow feature map is directly introduced into the neck network, a large amount of underlying noise will also be diffused into the entire feature fusion module, which will directly affect the feature fusion results of other detection layers. As a result, suppressing noise in the backbone network is of great significance for subsequent feature fusion. In addition, the feature extraction capability of the backbone network will also directly affect the final detection effect. These reasons make small target detection more dependent on efficient feature extraction networks. To suppress the background noise in the image, enhance the effective feature information of the target and allow the network to independently distinguish the correlation and effectiveness between different feature map channels to improve the detection effect, the triplet attention (TA) [47] module was introduced in this paper.

Spatial attention tells us where in the channel we should focus and channel attention tells us what channel we should focus on. Previously proposed attention mechanisms calculated channel attention and spatial attention separately; D. Misra [47] proposed the concept of cross-dimensional interaction, which emphasized the interaction between the spatial and channel dimensions of the input tensor. Triplet attention establishes interdimension dependencies through rotation and residual transformation, then re-encodes interchannel and spatial feature information and calculates the attention weight through the interdependence between the feature information dimensions, so that the network can provide a richer feature representation.

**Figure 4:**
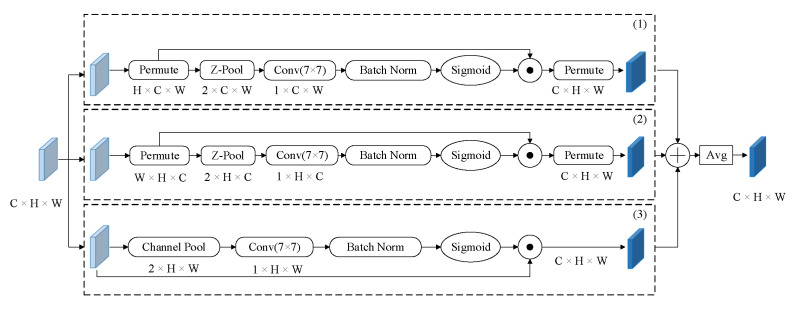
Illustration of the triplet attention, which has three branches. The first branch (1) is used to compute attention weights across channel dimension C and spatial dimension W. Similarly, the second branch (2) uses channel dimension C and spatial dimension H. The final branch (3) is used to capture spatial dependencies (H and W). Finally, the weights are aggregated by simple averaging.

Triplet attention consists of three parallel branches, two of which are used to capture the interaction between channel dimension and spatial dimension, and the third branch is used to calculate spatial attention; its structure is shown in Figure 4. Given an input feature map x $\in R^{C\times H\times W}$, branch (1) is used to compute the attention weights of channel dimension C and spatial dimension H. Firstly, the input x is rotated 90${}^{\circ }$ anticlockwise along the W axis and then performed a Z-pool operation. Secondly, a convolution layer with a kernel size of 7 × 7 and a batch normalization layer are used. Thirdly, it adopts a sigmoid activation layer to generate the attention weights. Finally, the attention weights retain the same input shape as x by rotation. Similarly, branch (2) computes the attention weight of channel dimension C and spatial dimension W like branch (1). For branch (3), the channels of x are reduced to two by the Z-pool layer, and then the space attention weights are computed by operations similar to the other two branches. In the end, the refined feature maps generated by the three branches are aggregated by simple averaging. The Z-pool layer here is responsible for reducing the zeroth dimension of the input feature map to two, by concatenating the average-pooled and max-pooled features across that dimension. Mathematically, it can be represented by Formula (2):

(2) $$\begin{array}{c} Z-\mathrm{Pool}\left(x\right)=\left[\mathrm{MaxPool}\left(x\right),\mathrm{AvgPool}\left(x\right)\right] \end{array}$$

**Figure 5:**
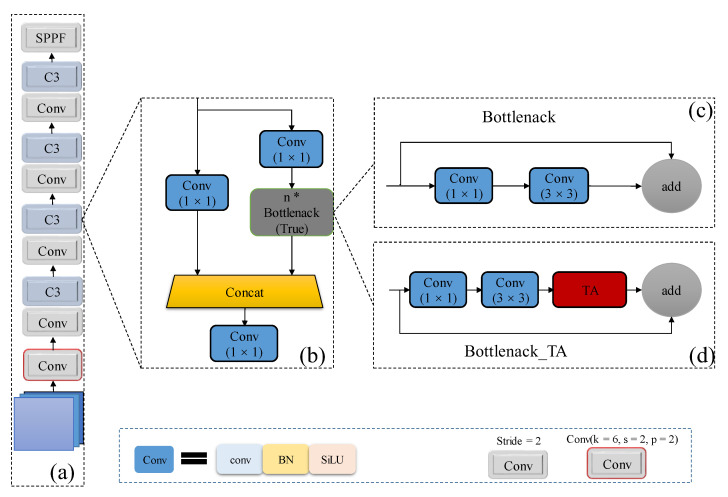
Illustration of the backbone with triplet attention. (**a**) Structure of backbone; (**b**) C3 module with residual structure; (**c**) Detailed structure of residual blocks in YOLOv5; (**d**) Proposed Bottleneck_TA module with triplet attention. * stands for repeating the module.

YOLOv5 uses CSPDarknet53 as the backbone network for multiscale feature extraction, which mainly consists of a convolution block with a convolution kernel of 6 × 6 and a stride of 2, three groups of Conv+C3 combined operations and a spatial pyramid pooling SPPF module, whose structure is shown in Figure 5a. The C3 module with residual block divides the feature map of the base layer into two parts to make the gradient flow propagate in different network paths by separating the gradient flow, and then the two parts are spliced through the cross-stage hierarchy; the structure is shown in Figure 5b. We introduced the TA attention mechanism into each C3 module in the backbone network by building a new Bottleneck_TA structure based on the original Bottleneck, as shown in Figure 5d. The TA is embedded in the backbone network, which can capture more effective feature information through spatial attention and channel attention cross-channel interaction, weakening the underlying noise interference brought by the P2 layer to a certain extent, which is beneficial to ship detection in infrared scenarios.

### 3.4. Feature Fusion

**Figure 6:**
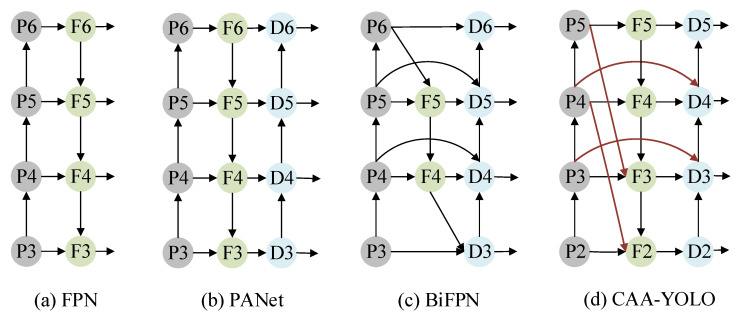
Feature network design: (**a**) FPN introduces a top-down pathway to fuse multiscale features from level 3 to 6 (P3–P6); (**b**) PANet adds an additional bottom-up pathway on top of FPN; (**c**) BiFPN with cross-scale connections (**d**) is our CAA-YOLO with more context information.

Four layers of feature maps are generated in the backbone network. Through these feature maps of different sizes, the neck network fuses feature maps of different levels to obtain more contextual information and reduce information loss. In the fusion process of YOLOv5, the feature pyramid structure of FPN and PAN is used. The FPN [21] structure transfers powerful semantic features from the top feature map to the lower one, as shown in Figure 6a. At the same time, the PAN [22] structure transfers strong localization features from the lower feature map to the higher ones, as shown in Figure 6b. To prevent the degradation of features between the same layers, BiFPN [11] introduces cross-layer connections to fuse more features without adding too much cost. Its structure is shown in Figure 6c.

Due to the weak features of infrared targets, especially small targets, the features are prone to degradation in the process of a continuous deepening of the convolutional network. Therefore, inspired by BiFPN, we introduced cross-layer connections between the same feature layers in YOLOv5, aiming to provide more features for the feature fusion stage. Furthermore, since the small target features in infrared ocean scenes are extremely weak, contextual information becomes extremely important for their detection process. Therefore, we added the paths from the feature layers P4 and P5 of the backbone network to the F2 and F3 layers based on YOLOv5, aiming to provide more contextual information for small targets. The structure is shown in Figure 6d.

The idea was as follows: On the one hand, because the target features smaller than 32 × 32 pixels of size have been highly compressed and even disappeared in the 32-time downsampling of P5, we did not introduce it to the F2 layer. On the other hand, the feature map P3, which was 8-time downsampled, was fused with F3 through horizontal connections and F3 was fused with F2 from top to bottom through the FPN network, so we also did not introduce the feature map P3 into the F2 layer. In the same way, we did not introduce the feature information of the P4 layer into the F3 layer.

**Figure 7:**
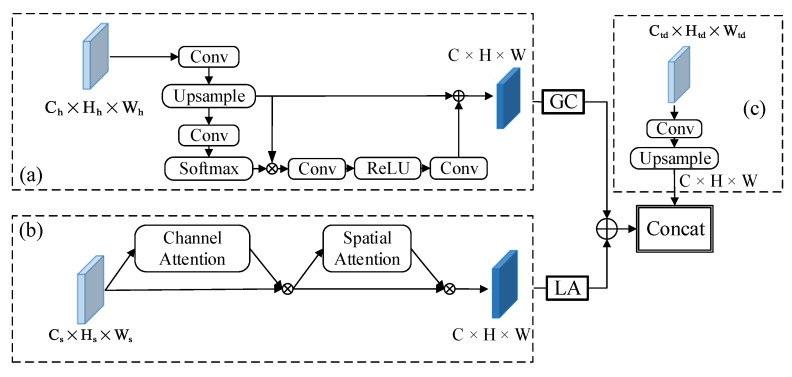
Illustration of the CAA module. (**a**) High-level feature maps use GC attention; (**b**) Low-level feature maps use CBAM attention; (**c**) Top-down feature information $F_{\mathrm{td}}$.

Because the shallow feature map P2 is directly fused with other multiscale feature layers, more noise will interfere with the feature fusion results. In addition, the feature fusion strategy used by YOLOv5 is to concatenate in terms of channel dimension. Concatenating features based on the channel dimension cannot reflect the correlation and importance of features between different channels. Without additional guidance, it is difficult for the underlying fusion module to accurately capture the key features of the target due to the interference of noise. Therefore, we needed to strengthen the feature fusion capabilities of the P2 and P3 layers for small target detection and suppress a large amount of noise interference at the bottom. The attention mechanism can flexibly capture the global and local relations in the input image, which focus on looking for important information related to the application task. We designed a hybrid attention module CAA, as shown in Figure 7. By improving the ability of the global context modeling and local feature extraction, the low-level small target detection layer can obtain more effective feature information. The CAA module mainly includes the information fusion of three feature maps, namely the high-level feature information $P_{h}$ from the backbone network, the same-layer feature information $P_{s}$ of the backbone network and the top-down feature information $F_{\mathrm{td}}$ in the FPN network.

For the high-level feature $P_{h}$ to fully utilize its contextual information, we adopted the attention module in GCNet [51] to capture long-range dependencies, whose structure is shown in Figure 7a. GCNet fully exploits the advantages and disadvantages of Non-Local [52] and SE [34] modules, absorbs the strong global context modeling ability of Non-Local Network (NLNet) and the low computational cost of SENet, and designs a more effective global context module to capture remote dependencies. Given an input tensor $P_{h}\in R^{C_{h}\times H_{h}\times W_{h}}$, first, it adjusts its channel to C × H × Wthrough a standard convolution operation with the BN layer and a 4-time downsampling layer and then passes it through the GC attention to capture more feature information.

For the low-level feature $P_{s}$ to make full use of the rich location information of the shallow feature map, the CBAM [53] attention module was used to selectively aggregate the features of each location by weighting the key location features. CBAM generates the attention map of the convolutional network feature map from two aspects of channel and space and then multiplies the attention map with the input feature map for feature adaptive learning. The network structure is shown in Figure 7b. The channel attention mechanism enables the network model to effectively pay attention to important channels while ignoring or even suppressing negative channels. The introduction of the spatial attention mechanism can effectively focus on the ship target and suppress other nonimportant information in the image, so as to further improve the detection accuracy.

After the high-level feature $P_{h}$ and the low-level feature $P_{s}$ were processed by the attention module, respectively, the feature fusion was performed by pixel-by-pixel addition. Then, it was combined with the top-down feature information $F_{\mathrm{td}}$ according to the dimension information to get the final feature fusion result. Through this module, important features can be enhanced and unimportant features can be weakened, so that the extracted features are more directional. Mathematically, it can be represented by Equation (3):

(3) $$\begin{array}{c} F^{\mathrm{out}}=\mathrm{Concat}\left[f_{\mathrm{sum}}\left(f_{\mathrm{CBAM}}\left(P_{s}\right),f_{\mathrm{GC}}\left(P_{h}\right)\right),f\left(F_{\mathrm{td}}\right)\right] \end{array}$$

$P_{s}$ is the underlying feature map in the backbone network, $P_{h}$ is the high-level feature map in the backbone network, $F_{\mathrm{td}}$ is the feature map in the top-down path, Concat[,] represents features that are spliced by channel, $f_{\mathrm{sums}}$ represent features that are pixel-by-pixel additively fused and $f_{\mathrm{CBAM}}$ and $f_{\mathrm{GC}}$ represent the feature enhancement operation before the feature layer fusion.

## 4. Experiments

In this section, we first introduce the experimental dataset, implementation details and related evaluation metrics. Furthermore, extensive experiments are also conducted to demonstrate the effectiveness and robustness of the proposed method.

### 4.1. Data Set

This paper used the infrared ship images provided by the organizers of the 2021 Ocean Target IntelliSense International Challenge as the data set, which contains a total of 9402 infrared images, including seven types of detection targets: warship, liner, container ship, bulk carrier, sailboat, canoe and fishing boat. We performed a preliminary manual screening of the dataset, removed some problematic images and readjusted the labels of some images to ensure the validity of the dataset.

**Figure 8:**
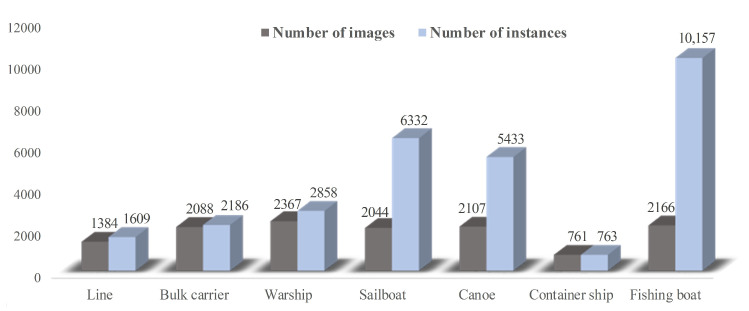
Data set distribution: The gray bar chart represents the number distribution of images for each type of ship; The blue bar chart represents the number distribution of each type of ship.

The distribution of each category of targets in the infrared ship data set is shown in Figure 8. The number of images and targets of fishing boats is approximately 1:5, which is more densely distributed than the other ships. After normalizing the size of the dataset to 640 × 640 pixels, the distribution of size and aspect ratio within the dataset are shown in Table 3 and Table 4, respectively. Table 3 shows the extreme difference in the size distribution of the datasets, with the smallest object being only 4 pixels and the largest reaching 408,960 pixels. Table 4 shows the variation between the target aspect ratios within the class. In total, 42% of the targets in the infrared ship dataset are small targets, whose size is less than 32 × 32 pixels, and are mainly distributed in fishing boat and canoe targets. Because small targets have few features and even present a dense distribution state, these problems further aggravate the difficulty of infrared small target detection.

### 4.2. Experimental Settings

#### 4.2.1. Implementation Details

The experiment environment was performed on a PC with Intel core i9-10900KF CPU, GeForce RTX 3090 (24 GB storage), CUDA 11.1 and the operating system was Ubuntu 18.04 LTS. The deep learning framework was Pytorch 1.9. Considering the equipment performance, the batch size was 24, and a total of 150 training epochs were selected with an initial learning rate of 0.01. The optimizer used stochastic gradient descent (SGD) and the cosine learning rate decay strategy to train the network. In our experiment, the images were resized to 640 × 640 uniformly. The dataset was split into approximately 80% training and 20% validation sets.

**Figure 9:**
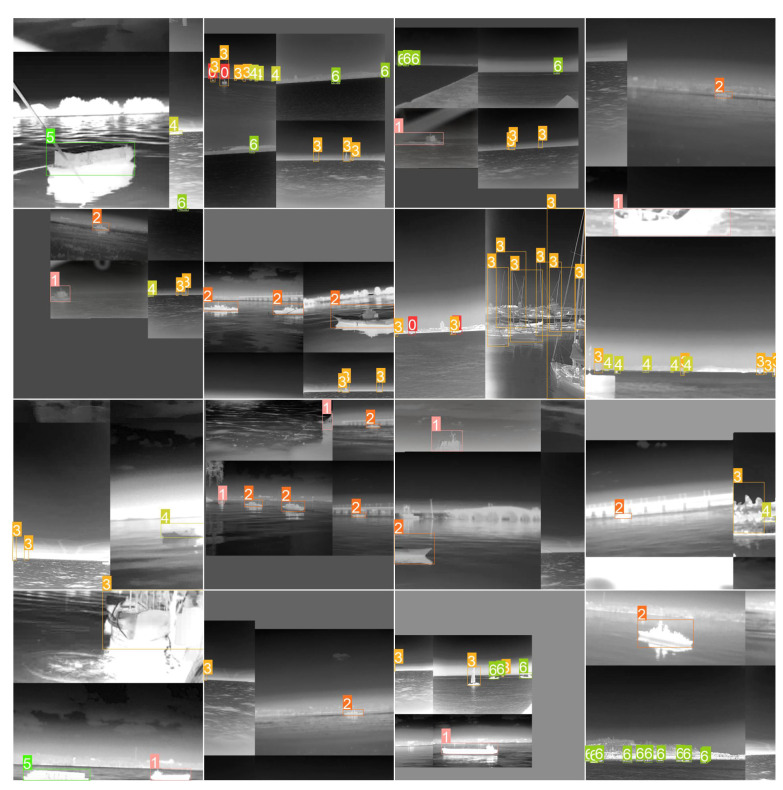
Mosaic data augmentation method. 0–6 denotes Liner, Bulk carrier, Warship, Sailboat, Canoe, Container ship, and Fishing boat, respectively.

We used the mosaic data augmentation method provided in Yolov5 to increase the number of small objects and increase the speed of training by stitching four images together, as shown in Figure 9.

#### 4.2.2. Evaluation Metrics

To evaluate the performance of the proposed algorithm, the classic detection metrics average precision (AP), average recall and mean average precision (mAP) were used. We calculated the AP and AR in two settings: AP@0.5/AR@0.5, AP@0.75/MR@0.75, APs/ARs, APm/ARm and APl/ARl. AP@0.5/AR@0.5 means the value of AP or AR when IoU = 0.5, AP@0.75/AR@0.75 means the value of AP or AR when IoU = 0.75, APs/ARs means the average value of AP or AR when the size of the target is smaller than 32 × 32 pixels, APm/ARm means the average value of AP or AR when the size of target is between 32 × 32 and 96 × 96 pixels, APl/ARl means the average value of AP or AR when the size of target is bigger than 96 × 96 pixels. Precision (P) and recall (R) were calculated by Formulas (4) and (5),

(4) $$\begin{array}{c} P=\frac{\#\mathrm{TP}}{\#\mathrm{TP}+\#\mathrm{FP}}\times 100\% \end{array}$$

(5) $$\begin{array}{c} P=\frac{\#\mathrm{TP}}{\#\mathrm{TP}+\#\mathrm{FN}}\times 100\% \end{array}$$

where # denotes the number, TP denotes the situation where the prediction and label are both ships, FP denotes the situation where the prediction is a ship but the label is the background, FN denotes the situation where the prediction is the background but the label is a ship. The average precision (AP) was calculated by Formula (6).

(6) $$\begin{array}{c} \mathrm{AP}=\int_{0}^{1}P\left(r\right)\cdot \mathrm{dr} \end{array}$$

where P denotes the precision, and r denotes the recall.

#### 4.2.3. Results

This section first introduces the experimental dataset, implementation details and related evaluation metrics. Furthermore, extensive experiments were also conducted to demonstrate the effectiveness and robustness of the proposed method.

(1) Influence of the P2 layer 

Because of a large number of small targets in the marine ship dataset, we aimed to use the feature information of small targets provided by the shallow layer P2 for classification and localization. We extracted the feature map downsampled four times from the backbone network as the P2 layer. It first performed feature fusion with the FPN, which transmitted high-level feature information from top to bottom, and then transferred the fused low-level features to the high-level detection layer through the PAN network. We set three anchor boxes of different scales on the high-resolution feature layer and obtained 12 sets of anchor boxes for the detection target by using the K-means algorithm, as shown in Table 5.

**Figure 10:**
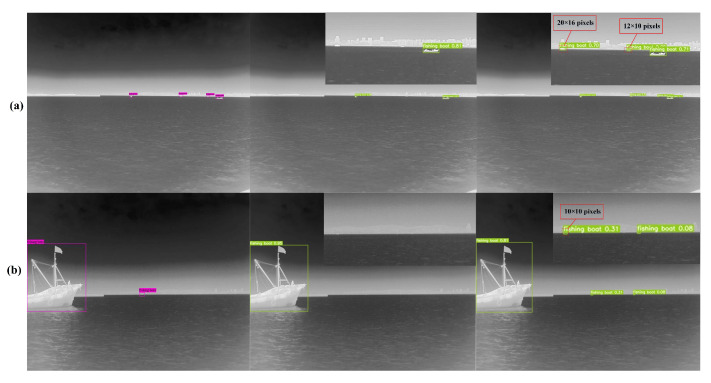
The detection results of small targets with different methods. GT represents the ground truth box, YOLOv5 represents the original baseline network model and YOLOv5+P2 represents the addition of high resolution feature layer P2 based on YOLOv5; (**a**,**b**) represent the 2 groups of detection results, respectively.

**Figure 11:**
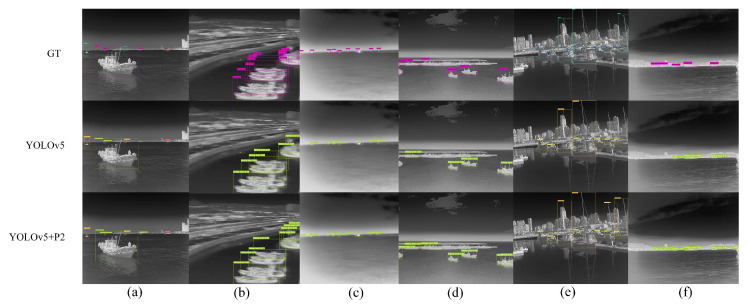
The infrared ship detection results of different methods. GT represents the ground truth box, YOLOv5 represents the original baseline network model and YOLOv5+P2 represents the addition of high resolution feature layer P2 based on YOLOv5; (**a**–**f**) represents the 6 groups of detection results obtained by using different models.

As shown in Figure 10, YOLOv5 missed the detection of targets with area smaller than 20 × 16 pixels, while adding the P2 layer could detect all small targets. Therefore, more low-level detailed information and positioning information can be extracted by adding the high-resolution feature layer P2, which is helpful to alleviate the phenomenon of missed detection of targets, especially for small targets. As shown in Figure 11a–c, our algorithm was able to detect small objects with extremely small size and weak features located on the sea line. In terms of the objects with indistinct features due to motion blur, such as Figure 11b,c, adding the P2 layer can achieve more accurate detection with more low-level details. In Figure 11d, ships occluding each other might compress the features due to downsampling, which often led to missed detection. However, adding the P2 layer also brought some noise interference. For example, Figure 11f showed error detection results. In Table 6, we find that the AP1 after adding P2 is lower than that of YOLOv5; the reason may be that the increase of the P2 layer at the bottom brings more noise to interfere with the result of the feature fusion at the top layers.

In conclusion, the addition of the P2 layer extracted more details of the targets, alleviated the missed detection of small targets and improved the detection rate of densely distributed targets. However, the detection of large targets was affected because the PAN network transmitted more noise introduced by P2 to the higher levels, so we had to suppress the shallow noise.

(2) Influence of the backbone 

By adding the P2 detection layer, we found that using the shallow feature could detect targets with weak features and small sizes, but it also brought more noise information to other detection layers. By introducing the TA attention network into the residual module of C3 in the backbone, we found that the noise increase could be alleviated to a certain extent, so that effective features could be assigned more weights to continue to propagate to the lower layers of the network and the propagation of invalid features could be suppressed. In Table 7, we find that the APl is improved relative to the model with the addition of P2 layers, which proves that the TA module can make the network pay more attention to the target area, thus reducing the influence of background on the result.

(3) Influence of the feature fusion 

**Figure 12:**
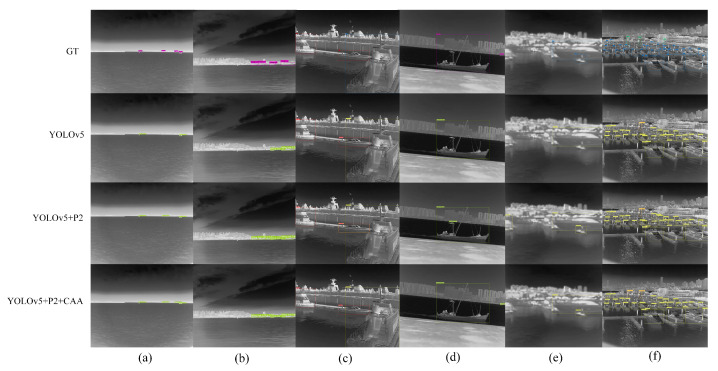
The infrared ship detection results of different methods. GT represents the ground truth box, YOLOv5 represents the original baseline network model, YOLOv5+P2 represents the addition of high resolution feature layer P2 based on YOLOv5, YOLOv5+P2+CAA represents the addition of P2 and the CAA feature fusion module based on YOLOv5 and (**a**–**f**) represents the 6 groups of detection results obtained by using different models.

The high-level feature layers P4 and P5 were used as context layers, respectively, and provided more contextual information for small objects through the proposed new feature fusion method. We applied an attention CAA module on the lower two layers to guide the fusion process of features and suppress noisy information. Figure 12a shows that the CAA module can detect more small targets. In Figure 12b–e, the addition of the P2 layer introduces more noise, which leads to the wrong detection of the target. The CAA module improves the detection. The group of images (Figure 12f) in the densely distributed scene reflect a better detection effectiveness of the CAA module. It can be seen from Table 5 and Table 7 that the average detection accuracy and recall rate of small targets improved, which were 3.39% and 6.67% higher than YOLOv5, and 2.41% and 5.01% higher than when only adding P2 layer. At the same time, the detection accuracy of large targets was also increased by 1.87% compared with the direct increase of the P2 layer, which solved the problem that the detection effectiveness of large targets decreased due to the addition of the P2 layer.

In conclusion, more context information was helpful for the target detection. The CAA fusion strategy improved the detection effectiveness of targets and suppressed the influence of the noise, which reduced false detection. Moreover, it also achieved a better detection effectiveness in the densely distributed scene and further improved the detection effectiveness of small targets, especially for targets below 10 × 10 pixels.

(4) Overall Detection Results 

**Figure 13:**
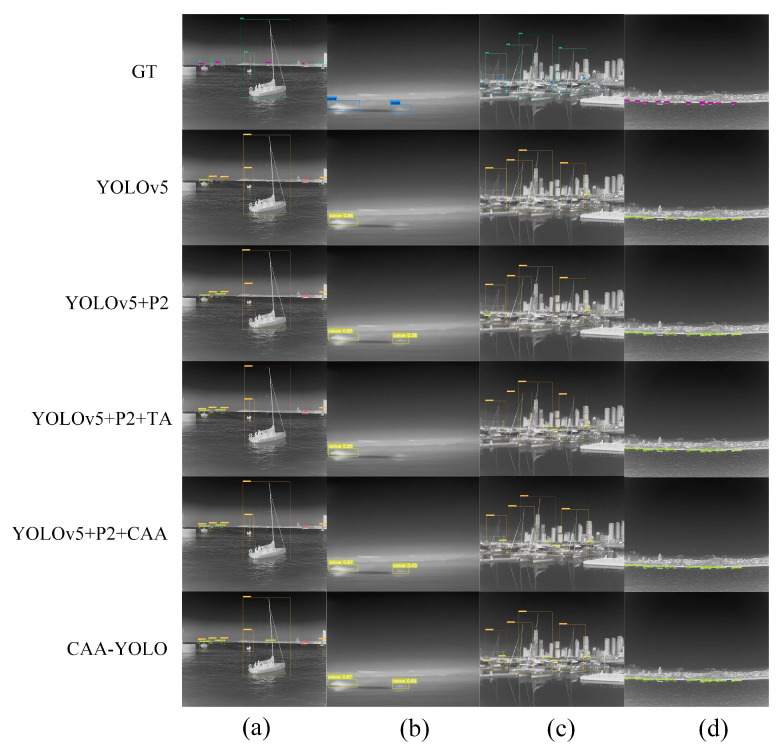
The infrared ship detection results of different methods. GT represents the ground truth box, YOLOv5 represents the original baseline network model, YOLOv5+P2 represents the addition of high resolution feature layer P2 based on YOLOv5, YOLOv5+P2+TA represents the addition of P2 layer and triple attention in backbone based on YOLOv5, YOLOv5+P2+CAA represents the addition of P2 and the CAA feature fusion module based on YOLOv5, CAA-YOLO represents the additions of all modules based on YOLOv5 and (**a**–**d**) represents the 4 groups of detection results obtained by using different models.

The algorithm proposed in this paper improved the detection effectiveness of small targets by constructing a high-resolution feature layer P2 and solved the noise interference caused by adding the P2 layer by means of a combined attention mechanism. By verifying the effectiveness of our algorithm in the infrared ocean dataset, it was found that CAA-YOLO could achieve more prominent detection results than YOLOv5 when facing extremely small targets. In addition, CAA-YOLO could also improve object detection in dense and occluded scenes. As shown in Figure 13a, our algorithm can not only detect extremely small targets on the water antenna, but also locate small targets in dense scenes. In Figure 13b, for targets with weaker features, our algorithm can also successfully detect targets, and the confidence of targets is improved by a combination of multiple strategies. In Figure 13c, our algorithm is able to detect more objects for complex and dense scenes. In Figure 13d, we are able to detect more small objects in the dense small object scene.

**Figure 14:**
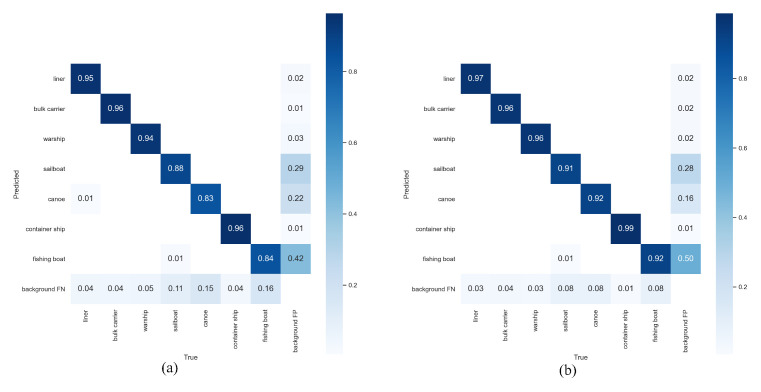
Confusion matrices. (**a**) YOLOv5; (**b**) CAA-YOLO.

**Figure 15:**
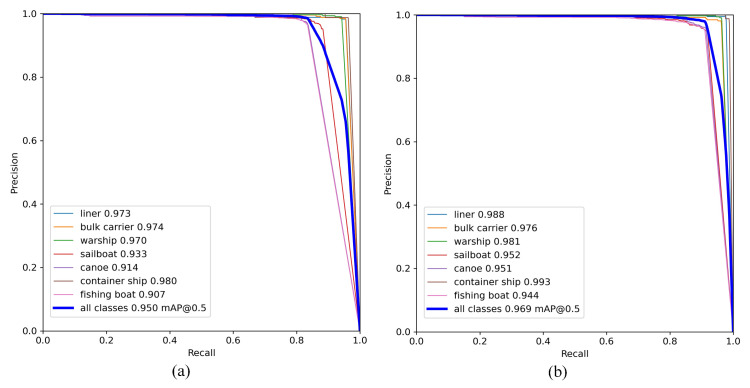
PR curve. (**a**) YOLOv5; (**b**) CAA-YOLO.

Compared with the YOLOv5 algorithm, the CAA-YOLO algorithm improved the detection effectiveness of each category, especially for smaller fishing boats and canoes, as shown in Figure 14. In general, our algorithm achieved better accuracy and recall rate, as shown in Figure 15. Although the average recall rate and average detection accuracy of the model improved less, the detection accuracy and recall rate of small targets were greatly improved, by 5.63% and 9.01%, respectively, as shown in Table 5 and Table 7. Compared with YOLOv5, our model had a 3.4% increase of mAP, a 5.81% increase in the number of parameters, and an increase in FLOPs from 108 to 131.9, as shown in Table 8.

As a result, our method could extract more target details without increasing computation too much, which is very important for occlusion scene and small target detection. In terms of time complexity, our GFLOPs increased by 23.9 and the increase in time complexity was mainly caused by the addition of the P2 layer. The CAA module brought only a small increase in floating point operations. In addition, our method hardly increased the space complexity of the model. For targets with drastic scale changes, CCA-YOLO could improve the recall rate and detection accuracy of small targets without reducing the detection effect of large targets.

(5) Comparison to State-of-the-Art Approaches 

To evaluate the effectiveness and timeliness of the proposed CAA-YOLO algorithm, we compared several state-of-the-art methods with our model, including faster R-CNN [4], SSD [8], RetinaNet [54] and EfficientDet [11]. For a fair comparison, all the compared methods adopted the same training and test sets and kept the same epoch of training. We tested the speed of image processing on a PC with Intel Xeon(R) CPU E5-2683 v3 CPU and GeForce GTX 1080 (12 GB storage).

Table 9 summarizes the detection results when IoU = 0.5 and confidence = 0.05 in terms of model volume, the mean average precision (mAP) and speed. ${\mathrm{mAP}}_{\mathrm{coco}}$ and ${\mathrm{mAP}}_{\mathrm{voc}}$ represent the results of detection using COCO and Pascal VOC evaluation methods, respectively. FPS (frame per second) is the detection speed, which is the number of images that the algorithm can detect per second. It can be seen that the proposed CAA-YOLO exhibits the best performance with the highest mAP values, which demonstrates its effectiveness compared with other models. Other algorithms show lower values in ${\mathrm{mAP}}_{\mathrm{coco}}$, but better performance in ${\mathrm{mAP}}_{\mathrm{voc}}$, indicating that their target positioning ability is weaker than that of YOLO and CAA-YOLO. When IoU is greater than 0.5, other advanced algorithms gradually show poor performance. Faster R-CNN can achieve better ${\mathrm{mAP}}_{\mathrm{voc}}$ compared with other compared methods, but its processing speed is slow because it is a two-stage algorithm. The SSD algorithm has the highest processing speed, but its detection performance is poor, especially for small targets.

**Figure 16:**
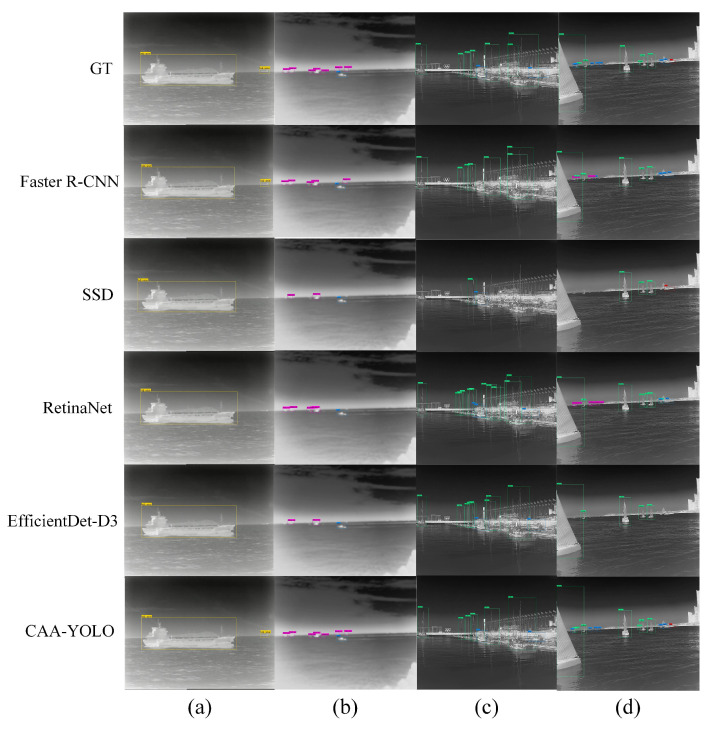
The infrared ship detection results of different methods. GT represents the ground truth box, the second to sixth lines represent the detection results of faster R-CNN, SSD, RetinaNet, EfficientDet-D3 and CAA-YOLO, respectively. (**a**–**d**) represents the 4 groups of detection results.

To further show the superior of CAA-YOLO visually, we present the detection results of several typical scenes in Figure 16. In Figure 16a, all algorithms can identify large targets correctly, but only faster R-CNN and CAA-YOLO algorithms can detect small targets with blurry visual effects on the sea surface. In Figure 16b, the target located at the sea line is small in size and weak in feature, the two-stage algorithm faster R-CNN can detect them better than the one-stage SSD, RetinaNet and EfficientDet, but it still misses targets, while CAA-YOLO can extract more target features to achieve a more accurate detection and alleviate missed detection. In Figure 16c, the ships near the shore are densely distributed and occluded from each other, and the detection effectiveness of faster R-CNN on canoes in complex backgrounds is poor. RetinaNet and EfficientDet have weak postprocessing capabilities for target occlusion scenes and fail to filter a large number of overlapping target boxes. CAA-YOLO can not only detect canoes in complex backgrounds, but also better detect densely distributed overlapping targets. In Figure 16d, other algorithms lead to error detection and missed detection of small targets, while CAA-YOLO can correctly detect more small targets. In these four examples, CAA-YOLO performs best, and the other four algorithms have different degrees of missed detection, false alarm and incomplete detection.

In conclusion, compared with several state-of-the-art methods, our proposed method obtained better target positioning ability and achieved real-time target detection performance. Because small targets have higher requirements for localization, CAA-YOLO showed better results in the detection of small targets.

## 5. Conclusions

In this paper, we proposed the CAA-YOLO network for infrared ocean ships detection to alleviate the problem of extremely widely multiscale distribution in infrared scenes. The network improved the detection effectiveness of small targets by adding a high-resolution detection layer and increased the feature extraction capability of the backbone by introducing an attention module. Furthermore, the combined attention mechanism used in the feature fusion stage could achieve a more effective feature fusion while suppressing infrared noise interference. We validated the effectiveness of the method using the infrared ship dataset and experiments illustrated substantial improvements in the infrared small target detection, in comparison with other state-of-the-art methods. The performance of our proposed model could be attributed to the combination of learned shallower features and attention features, which allowed our model to detect more infrared small targets based on their low resolution and weak features, and improved the target recall rate in dense and occluded scenes to a certain extent.

On the one hand, for dense and occluded objects, our algorithm could improve its recall rate, but there were still missed targets, so we will focus on solving the detection of dense and occluded targets in future work, for example, using better postprocessing mechanisms. On the other hand, compared with SSD and YOLOv5, we achieved better detection results, but the speed of detection was not the fastest, so we will further study how to lighten our method and improve its real-time detection speed. For example, depth-wise separable convolution and lighter backbones can be used to replace the backbone of our method.

## Figures and Tables

**Table 1:** Some related works based on deep learning in the field of infrared small target detection.

Strategy	Detection Algorithm	Description of Methods
Backbone	Lin et al. [32]	A 7-layer CNN was designed to automatically extract small target features and suppress clutters in an end-to-end manner.
	SE-YOLO [33]	An SE block was introduced into YOLOv3 to achieve higher accuracy and lower false alarm rate in small pedestrian detection task.
	YOLO-ACN [35]	An attention mechanism was introduced in the channel and spatial dimensions in each residual block of YOLOv3 to focus on small targets.
	I-YOLO [36]	A dilated-residual U-Net was also introduced to reduce the noise of infrared road images.
	FA-YOLO [38]	A dilated CBAM module was added to the CSPDarknet53 in the YOLOv4 backbone.
	TIRNet [37]	A residual branch was added when training to force the network to learn robust and discriminating features.
Fusion strategy	ACM-U-Net [39]	An asymmetric contextual modulation module was proposed for detecting infrared small targets based on FPN and U-Net [46].
	DNANet [44]	A DNIM module was designed to achieve progressive interaction among high-level and low-level features on a infrared small target dataset.
	ThermalDet [45]	A DFB block and CEM module were designed to directly fuse features from all different levels based on RefineDet.
High-resolution detection layer	YOLO-FIRI [1]	Multiscale detection was added to improve small object detection accuracy.

**Table 2:** The distribution of small targets in the infrared ocean dataset.

Area (pix)			
Type (Number)	⩽10 × 10	⩽16 × 16	⩽32 × 32
Liner	0	29	258
Bulk carrier	0	17	175
Warship	23	139	625
Sailboat	3	76	560
Canoe	87	423	1239
Container ship	0	0	44
Fishing boat	229	1460	5765

**Table 3:** The size distribution of the targets. The max, min, mean variance and S/M/L, respectively, represent the maximum area, the minimum area, the mean value of the area, the variance of the area and the number of small, medium and large targets in one class of ships.

Ship Type	Max	Min	Mean	Variance	S/M/L
Liner	306,600	36	21,817	1,330,938,169	602/266/741
Bulk carrier	408,960	40	36,785	2,703,590,022	164/562/1460
Warship	308,016	195	16,758	672,915,660	278/1191/1389
Sailboat	384,678	20	19,207	1,197,080,051	1800/2044/2488
Canoe	279,000	6	7567	421,559,886	2020/2354/1059
Container ship	122,808	216	14,565	210,081,332	115/284/364
Fishing boat	408,321	4	2278	190,665,384	8249/1020/888

**Table 4:** The aspect ratio distribution of the target. The max, min, mean and variance, respectively, represent the maximum, minimum, mean and variance of the target aspect ratio in one class of ships.

Area (pix)				
Ship Type	Max	Min	Mean	Variance
Liner	6.73	0.23	1.76	0.72
Bulk carrier	14.7	0.19	2.43	1.57
Warship	4.28	0.24	2.16	0.56
Sailboat	3.82	0.05	0.43	0.04
Canoe	7.48	0.18	1.59	0.53
Container ship	5.62	0.35	2.87	1.47
Fishing boat	15.5	0.11	2.06	0.79

**Table 5:** Anchor of each detect head.

Detection Layer	Anchor
D2	(10,5), (15,22), (21,7)
D3	(28,14), (37,57), (53,23)
D4	(49,130), (99,39), (188,54)
D5	(106,202), (189,273), (300,97)

**Table 6:** Average precision of experimental results when IoU = 0.6 and confidence = 0.3.

Model	AP (%)	AP50 (%)	AP75 (%)	APs (%)	APm (%)	APl (%)
YOLOv5	73.79	91.61	81.83	44.96	74.07	85.54
YOLOv5+P2	74.3	93.37	83.46	45.94	75.59	83.96
YOLOv5+P2+TA	74.37	93.83	83.87	46.12	75.28	85.56
YOLOv5+P2+CAA	74.79	93.84	84.14	48.35	75.63	85.83
CAA-YOLO	75.35	94.25	83.98	50.59	76.16	87.31

**Table 7:** Average recall of experimental results when IoU = 0.6 and confidence = 0.3.

Model	AR (%)	AR50 (%)	AR75 (%)	ARs (%)	ARm (%)	ARl (%)
YOLOv5	55.45	76.06	76.92	50.06	77.84	87.26
YOLOv5+P2	55.76	77.29	78.38	51.72	79.95	88.32
YOLOv5+P2+TA	55.93	77.47	78.84	56.73	79.96	88.33
YOLOv5+P2+CAA	55.97	77.81	78.84	56.73	79.96	88.33
CAA-YOLO	56.98	78.22	79.31	59.07	80.33	89.78

**Table 8:** Experimental results of different optimization strategies.

Model	mAP@0.5:0.95 (%)	GFLOPs	Params (MB)
YOLOv5	79.4	108	92.9
YOLOv5+P2	79.8	127.5	95.5
YOLOv5+P2+TA	80.1	129.2	95.8
YOLOv5+P2+CAA	81.5	130.1	98
CAA-YOLO	82.8	131.9	98.3

**Table 9:** Detection results of different algorithms on the infrared ship dataset.

Model	Framework	${\mathrm{mAP}}_{\mathrm{coco}}$	${\mathrm{mAP}}_{\mathrm{voc}}$	Params (MB)	FPS
Faster-RCNN	ResNet50+FPN	59.74	86.97	166	20
SSD	ResNet50+FPN	48.21	74.47	55.8	100
RetinaNet	ResNet50+FPN	46.82	79.19	146	22
EfficientDet-D3	EfficientNet+BiFPN	46.17	80.65	48.5	14
YOLOv5	CSPDarknet+PAN	79.40	90.09	92.9	53
CAA-YOLO	CAA-YOLO	82.8	94.81	98.3	42

## Data Availability

Not applicable.

## References

[1] Li S., Li Y., Li Y., Li M., Xu X. (2021). YOLO-FIRI: Improved YOLOv5 for Infrared Image Object Detection. IEEE Access.

[2] Girshick R., Donahue J., Darrell T., Malik J. Rich feature hierarchies for accurate object detection and semantic segmentation. Proceedings of the IEEE Conference on Computer Vision and Pattern Recognition.

[3] Girshick R. Fast r-cnn. Proceedings of the IEEE International Conference on Computer Vision.

[4] Ren S., He K., Girshick R., Sun J. Faster r-cnn: Towards real-time object detection with region proposal networks. Proceedings of the 28th International Conference on Neural Information Processing Systems.

[5] Redmon J., Divvala S., Girshick R., Farhadi A. You only look once: Unified, real-time object detection. Proceedings of the IEEE Conference on Computer Vision and Pattern Recognition.

[6] Redmon J., Farhadi A. (2018). YOLOv3: An Incremental Improvement. arXiv.

[7] Bochkovskiy A., Wang C.Y., Liao H.Y.M. (2020). Yolov4: Optimal speed and accuracy of object detection. arXiv.

[8] Liu W., Anguelov D., Erhan D., Szegedy C., Reed S., Fu C.Y., Berg A.C. (2016). Ssd: Single shot multibox detector. European Conference on Computer Vision.

[9] Nayan A.A., Saha J., Mozumder A.N., Mahmud K.R., Al Azad A.K. (2020). Real Time Detection of Small Objects Detection and Recognition Using Vision Augmentation Algorithm. arXiv.

[10] Chen C., Liu M.Y., Tuzel O., Xiao J. (2016). R-CNN for small object detection. Asian Conference on Computer Vision.

[11] Tan M., Pang R., Le Q.V. Efficientdet: Scalable and efficient object detection. Proceedings of the IEEE/CVF Conference on Computer Vision and Pattern Recognition.

[12] Lim J.S., Astrid M., Yoon H.J., Lee S.I. Small object detection using context and attention. Proceedings of the 2021 International Conference on Artificial Intelligence in Information and Communication (ICAIIC).

[13] Zhang Y., Guo L., Wang Z., Yu Y., Liu X., Xu F. (2020). Intelligent ship detection in remote sensing images based on multi-layer convolutional feature fusion. Remote Sens..

[14] Kim M., Jeong J., Kim S. (2021). ECAP-YOLO: Efficient Channel Attention Pyramid YOLO for Small Object Detection in Aerial Image. Remote Sens..

[15] Shao J., Yang Q., Luo C., Li R., Zhou Y., Zhang F. (2021). Vessel Detection From Nighttime Remote Sensing Imagery Based on Deep Learning. IEEE J. Sel. Top. Appl. Earth Obs. Remote Sens..

[16] Bi F., Hou J., Chen L., Yang Z., Wang Y. (2019). Ship detection for optical remote sensing images based on visual attention enhanced network. Sensors.

[17] Cui Z., Wang X., Liu N., Cao Z., Yang J. (2020). Ship detection in large-scale SAR images via spatial shuffle-group enhance attention. IEEE Trans. Geosci. Remote Sens..

[18] Chen L., Shi W., Deng D. (2021). Improved YOLOv3 based on attention mechanism for fast and accurate ship detection in optical remote sensing images. Remote Sens..

[19] Dewi C., Chen R.C., Jiang X., Yu H. (2022). Deep convolutional neural network for enhancing traffic sign recognition developed on Yolo V4. Multimed. Tools Appl..

[20] Liu S., Huang D., Wang Y. Receptive field block net for accurate and fast object detection. Proceedings of the European Conference on Computer Vision (ECCV).

[21] Lin T.Y., Dollár P., Girshick R., He K., Hariharan B., Belongie S. Feature pyramid networks for object detection. Proceedings of the IEEE Conference on Computer Vision and Pattern Recognition.

[22] Liu S., Qi L., Qin H., Shi J., Jia J. Path aggregation network for instance segmentation. Proceedings of the IEEE Conference on Computer Vision and Pattern Recognition.

[23] Zhou P., Ni B., Geng C., Hu J., Xu Y. Scale-transferrable object detection. Proceedings of the IEEE Conference on Computer Vision and Pattern Recognition.

[24] Jiang J., Fu X., Qin R., Wang X., Ma Z. (2021). High-speed lightweight ship detection algorithm based on YOLO-v4 for three-channels RGB SAR image. Remote Sens..

[25] Hu J., Zhi X., Shi T., Zhang W., Cui Y., Zhao S. (2021). PAG-YOLO: A portable attention-guided YOLO network for small ship detection. Remote Sens..

[26] Rezatofighi H., Tsoi N., Gwak J., Sadeghian A., Reid I., Savarese S. Generalized intersection over union: A metric and a loss for bounding box regression. Proceedings of the IEEE/CVF Conference on Computer Vision and Pattern Recognition.

[27] Zheng Z., Wang P., Liu W., Li J., Ye R., Ren D. Distance-IoU loss: Faster and better learning for bounding box regression. Proceedings of the AAAI Conference on Artificial Intelligence.

[28] Kisantal M., Wojna Z., Murawski J., Naruniec J., Cho K. (2019). Augmentation for small object detection. arXiv.

[29] Chen C., Zhang Y., Lv Q., Wei S., Wang X., Sun X., Dong J. Rrnet: A hybrid detector for object detection in drone-captured images. Proceedings of the IEEE/CVF International Conference on Computer Vision Workshops.

[30] Gadekallu T.R., Srivastava G., Liyanage M., Iyapparaja M., Chowdhary C.L., Koppu S., Maddikunta P.K.R. (2022). Hand gesture recognition based on a Harris hawks optimized convolution neural network. Comput. Electr. Eng..

[31] Loshchilov I., Hutter F. (2016). Sgdr: Stochastic gradient descent with warm restarts. arXiv.

[32] Liangkui L., Shaoyou W., Zhongxing T. (2018). Using deep learning to detect small targets in infrared oversampling images. J. Syst. Eng. Electron..

[33] Li M., Zhang T., Cui W. (2020). Research of infrared small pedestrian target detection based on YOLOv3. Infrared Technoiogy.

[34] Hu J., Shen L., Sun G. Squeeze-and-excitation networks. Proceedings of the IEEE Conference on Computer Vision and Pattern Recognition.

[35] Li Y., Li S., Du H., Chen L., Zhang D., Li Y. (2020). YOLO-ACN: Focusing on small target and occluded object detection. IEEE Access.

[36] Sun M., Zhang H., Huang Z., Luo Y., Li Y. (2022). Road infrared target detection with I-YOLO. IET Image Process..

[37] Dai X., Yuan X., Wei X. (2021). TIRNet: Object detection in thermal infrared images for autonomous driving. Appl. Intell..

[38] Du S., Zhang B., Zhang P., Xiang P., Xue H. (2021). FA-YOLO: An Improved YOLO Model for Infrared Occlusion Object Detection under Confusing Background. Wirel. Commun. Mob. Comput..

[39] Dai Y., Wu Y., Zhou F., Barnard K. Asymmetric contextual modulation for infrared small target detection. Proceedings of the IEEE/CVF Winter Conference on Applications of Computer Vision.

[40] Zhang J., Jin Y., Xu J., Xu X., Zhang Y. (2018). Mdu-net: Multi-scale densely connected u-net for biomedical image segmentation. arXiv.

[41] Dolz J., Ben Ayed I., Desrosiers C. (2018). Dense multi-path U-Net for ischemic stroke lesion segmentation in multiple image modalities. International MICCAI Brainlesion Workshop.

[42] Huang H., Lin L., Tong R., Hu H., Zhang Q., Iwamoto Y., Han X., Chen Y.W., Wu J. Unet 3+: A full-scale connected unet for medical image segmentation. Proceedings of the ICASSP 2020—2020 IEEE International Conference on Acoustics, Speech and Signal Processing (ICASSP).

[43] Zhou Z., Siddiquee M.M.R., Tajbakhsh N., Liang J. (2019). Unet++: Redesigning skip connections to exploit multiscale features in image segmentation. IEEE Trans. Med. Imaging.

[44] Li B., Xiao C., Wang L., Wang Y., Lin Z., Li M., An W., Guo Y. (2021). Dense nested attention network for infrared small target detection. arXiv.

[45] Cao Y., Zhou T., Zhu X., Su Y. Every feature counts: An improved one-stage detector in thermal imagery. Proceedings of the 2019 IEEE 5th International Conference on Computer and Communications (ICCC).

[46] Ronneberger O., Fischer P., Brox T. (2015). U-net: Convolutional networks for biomedical image segmentation. International Conference on Medical Image Computing and Computer-Assisted Intervention.

[47] Misra D., Nalamada T., Arasanipalai A.U., Hou Q. Rotate to attend: Convolutional triplet attention module. Proceedings of the IEEE/CVF Winter Conference on Applications of Computer Vision.

[48] Shrivastava A., Gupta A. (2016). Contextual priming and feedback for faster r-cnn. European Conference on Computer Vision.

[49] Cai Z., Fan Q., Feris R.S., Vasconcelos N. (2016). A unified multi-scale deep convolutional neural network for fast object detection. European Conference on Computer Vision.

[50] Sermanet P., Kavukcuoglu K., Chintala S., LeCun Y. Pedestrian detection with unsupervised multi-stage feature learning. Proceedings of the IEEE Conference on Computer Vision and Pattern Recognition.

[51] Cao Y., Xu J., Lin S., Wei F., Hu H. Gcnet: Non-local networks meet squeeze-excitation networks and beyond. Proceedings of the IEEE/CVF International Conference on Computer Vision Workshops.

[52] Wang X., Girshick R., Gupta A., He K. Non-local neural networks. Proceedings of the IEEE Conference on Computer Vision and Pattern Recognition.

[53] Woo S., Park J., Lee J.Y., Kweon I.S. Cbam: Convolutional block attention module. Proceedings of the European Conference on Computer Vision (ECCV).

[54] Lin T.Y., Goyal P., Girshick R., He K., Dollár P. Focal loss for dense object detection. Proceedings of the IEEE International Conference on Computer Vision.

